# Maladie de Bowen du pied droit: Bowen’s disease of the right foot

**DOI:** 10.11604/pamj.2018.29.3.14441

**Published:** 2018-01-02

**Authors:** Youssef Zemmez, Mohammed Boui

**Affiliations:** 1 Service de Dermatologie, Hôpital Militaire Mohamed V, Rabat, Maroc

**Keywords:** Bowen’s disease, histology, surgery, Maladie de Bowen, histologie, chirurgie

## Abstract

We here report the case of a16-year old patient, with no particular past medical history, presenting, with skin swelling at the level of the forefoot evolving for 02 years, at the Department of Dermatology. Clinical examination showed hard budding cutaneous mass painless on palpation covering the 2^nd^, the 3^rd^ and the 4^th^, right toes, measuring 4 cm in large diameter. The remainder of the clinical examination was normal, without adenopathies. Skin biopsy was performed at the level of the tumor and histological examination showed carcinoma in situ. Surgical treatment was recommended. Bowen’s disease is a relatively rare squamous intraepithelial carcinoma in situ. It mainly affects adults at any age, with predominance in women. The diagnosis is suspected on the basis of clinical examination, but it is confirmed by the anatomo-pathological examination.

## Image en médecine

Patiente âgée de 16 ans, sans antécédents pathologiques notables, qui a consulté en dermatologie pour une tuméfaction cutanée siégeant au niveau de l'avant pied droit évoluant depuis 02 ans. L'examen clinique a objectivé une masse cutanée bourgeonnante de consistance dure, indolore à la palpation couvrant les 2^ème^, 3^ème^ et 4^ème^ orteils droits, mesurant 4 cm de grand diamètre. Le reste de l'examen clinique était normal notamment pas d'adénopathies. Une biopsie cutanée a été réalisée au niveau de la tumeur dont l'histologie était en faveur d'un carcinome in situ. Un traitement chirurgical a été indiqué. La maladie de Bowen est un carcinome in situ malpighien intraépithélial relativement rare. Elle atteint surtout l'adulte à tout âge avec une prédominance féminine. Le diagnostic est suspecté cliniquement, mais c'est l'examen anatomopathologique qui le confirme.

**Figure 1:**
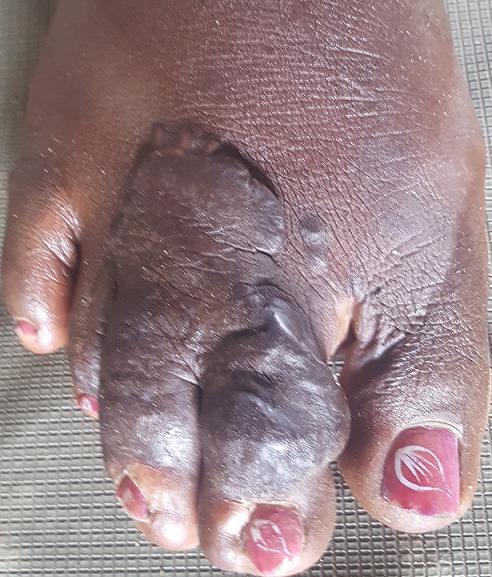
maladie de Bowen du pied droit

